# *Acanthamoeba polyphaga mimivirus* and other giant viruses: an open field to outstanding discoveries

**DOI:** 10.1186/1743-422X-11-120

**Published:** 2014-06-30

**Authors:** Jônatas S Abrahão, Fábio P Dornas, Lorena CF Silva, Gabriel M Almeida, Paulo VM Boratto, Phillipe Colson, Bernard La Scola, Erna G Kroon

**Affiliations:** 1 Universidade Federal de Minas Gerais, Instituto de Ciências Biológicas, Laboratório de Vírus, Avenida Antônio Carlos, 6627, Caixa Postal 486, Bloco F4, Sala 258, 31270-901 Belo Horizonte, Minas Gerais, Brazil; 2 URMITE, UM63, CNRS 7278, IRD 198, Inserm 1095, Aix Marseille Universite, Marseille, France

**Keywords:** Giant viruses, *Mimiviridae*, *Mimivirus*

## Abstract

In 2003, *Acanthamoeba polyphaga mimivirus* (APMV) was first described and began to impact researchers around the world, due to its structural and genetic complexity. This virus founded the family *Mimiviridae*. In recent years, several new giant viruses have been isolated from different environments and specimens. Giant virus research is in its initial phase and information that may arise in the coming years may change current conceptions of life, diversity and evolution. Thus, this review aims to condense the studies conducted so far about the features and peculiarities of APMV, from its discovery to its clinical relevance.

## Introduction

Viruses are remarkable organisms that have always attracted scientific interest. The study of unique viral features has been a wellspring of discovery that helped establish the foundations of molecular biology and led to in-depth evolutionary studies [1-3]. In this context, giant viruses have recently emerged as a fascinating line of research, raising important questions regarding evolution and their relationships with their hosts [4-10].

Although giant viruses offer deep ecological and clinical importance, the *Mimiviridae* group deserves special emphasis; they have been the subject of intense research in recent years, which has generated much relevant information [4-8,11-14]. *Acanthamoeba polyphaga mimivirus* (APMV) was the first known mimivirus, isolated from an amoebal co-culture present in a water sample collected from a cooling tower of a hospital in England. Its characterization revealed surprising characteristics: it was a DNA virus with a diameter of approximately 700 nm and a genome of approximately 1,2 Mb, making it the largest known virus up to then [5]. Five years later, a similar virus was isolated in amoeba from the water of a cooling tower in Paris, and since then several dozen giant viruses have been isolated from many different environments and specimens [11,15-19]. Data obtained from these viruses provoked scientific discussions regarding the nature and biological dynamics of viruses, and the intriguing features *Mimivirus* have challenged virologists and evolutionists alike. The potential existence of many other interesting and unusual unknown viruses in the biosphere makes us realize that the discovery and characterization of giant viruses is in its initial phase and that there is still much to be learned by studying these organisms. Recently, the discovery of Pandoravirus (1 μm in length and with a genome fo 2.8 Mb) and of *Pithovirus sibericum* (1.5 μm in length and a surprisingly smaller genome of 600 kb) brought even more attention to the prospection and study of giant viruses [19,20].

## Discovery and taxonomy

In 1992, a pneumonia outbreak occurred in a Bradford hospital (England), and water samples from a cooling tower that contained free-living amoebae were investigated to determine the etiological agent of the pneumonia outbreak [5]. At that time, Gram-positive cocci that were visualized by light microscopy inside *Acanthamoeba polyphaga* cells were named Bradford coccus. Every attempt to isolate this microorganism and amplify its 16S rDNA failed. Moreover, treatment of amoeba cultures with antibiotics to inhibit growth of this microorganism was also unsuccessful, which led doubt whether it was indeed a bacteria [5]. After a hiatus of a few years, this organism was the subject of new studies at the *Rickettsia Unit at the School of Medicine* (Marseille, France) in the early 2000s. After a new series of unsuccessful characterization attempts, electron microscopy of *Bradfordcoccus*-infected *Acanthamoeba polyphaga* cells revealed icosahedral-like particles with an astonishing 750 nm diameter size [5]. In addition to this virus-like morphology, analysis of the replication curve of this organism in amoeba cells revealed an eclipse phase, which is an almost universal feature among viruses. Finally, when the complete sequencing and analysis of its genome was finished, it became evident that this peculiar organism clustered with other giant viruses and not with bacteria [5]. This new virus was then called *Acanthamoeba polyphaga* mimivirus (APMV), due its ability to infect the free-living amoebae *Acanthamoeba polyphaga* sp. and mimic a microbe. APMV, also known as mimivirus, had the largest viral genome known up to then, reaching approximately 1.2 Mb. Its characteristics were so different from other viruses that it was not possible to include it into any known viral family, so the *Mimiviridae* family was created [5]. APMV became the first member of the *Mimiviridae* family, *Mimivirus* genus, and its prototype.

Few years before the discovery of APMV, the genomic content of known giant viruses that replicate partly or entirely in the cytoplasm of eukaryotic cells (members from *Poxviridae*, *Asfarviridae*, *Phycodnaviridae, Ascoviridae* and *Iridoviridae* families) was analyzed in depth. Several common, and supposedly essential, genes were identified, and it was suggested that the origin of these four viral families was monophyletic [21-26]. Other common characteristics, such as a large double-stranded DNA genome, the relative independence of their host transcription machinery, and a replication cycle that occurs at least partially into the cytoplasm with the formation of inclusion bodies or viral factories, were the basis for the generic name of these viral families: the nucleo-cytoplasmic large DNA viruses (NCLDVs) [22-26]. Because viruses from the *Mimiviridae* family also share these characteristics, they were added to the NCLDV group as well.

After APMV was described, interest in giant viruses grew, several other giant viruses were isolated, and the *Mimiviridae* family was expanded. Currently, the *Mimiviridae* family contains two genera according to the International Committee on Taxonomy of Viruses (ICTV): *Mimivirus*, with APMV as its only member, and *Cafeteriavirus*, with *Cafeteria roenbergensis virus* as its only member (http://www.ictvonline.org). With the increasing number of new giant virus isolates and hypothetical species, a new viral order has been proposed [27]. The putative *Megavirales* order contains the *Mimiviridae* family and other NCLDVs, including the newly proposed *Marseilleviridae* family [28], whose founding member is Marseillevirus, another giant virus, smaller than Mimivirus, that infects amoeba isolated from cooling tower water in 2008 [18]. Mimivirus and Marseillevirus have been primarily linked to other NCLDVs, based on a set of ≈ 50 conserved core genes shared by all or by a majority of these large and giant viruses [29]. All these viruses were shown to compose a monophyletic group [21]. Nucleo cytoplasmic virus orthologous groups (NCVOGs) were defined among these viruses, including 177 proteins present in > 1 NCLDV family and five common to all viruses [30].

The discovery of new isolates of mimiviruses of amoeba revealed the existence of three different lineages (A, B and C). Lineage A of the *Mimivirus* genus contains the best known mimivirus isolates, such as the APMV species [5]. *Mimivirus* lineage B is represented by *Acanthamoeba polyphaga* moumouvirus, which was isolated from a water sample in February, 2008; genetic analysis revealed differences that placed this virus into the B lineage [15]. The first extensively described member of lineage C was *Megavirus chilensis*, isolated from a water sample collected off the coast of Chile [13]. Other previously described mimiviruses, including Courdo7, Courdo11, Terra1 and Montpellier, were also included in lineage C [31]. Other viruses, including some that are distantly related, have been obtained from environmental and clinical samples [32,33], and in the next years it will likely be possible to present an extensive depiction of the putative viral isolates, strains and species.

## Amoebas as the main host of giant viruses

As mentioned above, the first mimivirus isolate was discovered from studies of the pathogenic microorganisms associated with amoebae (MPAAs) that were linked to nosocomial pneumonia [5]. Free-living amoebae of the genus *Acanthamoeba* belong to the *Protist* kingdom and can be part of the normal microbiota of some animals, including humans [34]. Amoebas from this genus are considered ubiquitous and have been isolated from various environments, including soil, air, aquatic environments, sewage treatment systems, contact lenses, hospital environments, and ventilation and air conditioning systems [34-36]. Several studies show that amoebas of this genus are very stable after treatment with disinfectants and are highly resistant to extremes of pH and temperature [34-37].

Free-living amoebae can cause severe and chronic diseases by themselves, such as granulomatous amoebic encephalitis, cutaneous acanthamoebiasis, amoebic keratitis and primary amoebic meningoencephalitis [38]. They can also carry other MPAAs that cause disease [39-41]. For example, microorganisms of the *Legionella*, *Parachlamydia* and *Mycobacterium* genera are MPAAs considered to be causative agents of pneumonia, most of which is associated with many cases of nosocomial lung infection [37,42,43]. Even after being phagocytosed, some MPAAs are able to persist in the amoeba intracellular environment, and often manage to multiply numerously [5]. Studies have described amoebae isolation from many health institutions, revealing the presence of free living amoebae on hospital floors and objects, in intensive care units (ICUs), operating rooms, nurseries, kitchens, emergency rooms and infectious disease wards, showing that the free living amoebas may serve as potential platforms for amplification of pathogenic MPAAS in these environments [40-43]. The attention given to free living amoebas in recent years, together with the isolation and characterization of mimiviruses in amoebal samples from a cooling tower during an outbreak of pneumonia, add more importance to the role of mimiviruses as MPAAs.

Up to now, amoebae of the *Acanthamoeba* genus are the only confirmed APMV hosts; the virus was originally isolated from *A. polyphaga*, but now is also being cultivated in the laboratory in *A. castelannii*, *A. griffin* and *A. lenticulata*[5,44]. However, there is increasing evidence that these viruses have a broader host range. Some studies have indicated sponges and corals as potential hosts of mimivirus [44]. Khan (2007) described a productive APMV infection in mice after intracardiac infection [45]. The ability of APMV to enter and replicate inside human phagocytic cells and peripheral blood mononuclear cells in vitro has been reported [39]. These reports, together with the human blood isolation of the marseillevirus, another giant virus of amoeba belonging to a different family, suggests that vertebrates may also be hosts of these viruses [46]. The mimivirus genome has been detected in monkeys and bovines, supporting these findings [47,48]. Recently, it has been shown that APMV is able to interact with the human interferon system, a strong clue that both species share an evolutionary history [49]. It appears that APMV’s ability to enter cells by phagocytosis without identified specific cell receptors, together with its large genome that confers a powerful array of non-essential genes, permits APMV to exploit a larger host range than initially believed [39,49]. Lastly, as described in this review, there is ample evidence associating APMV and other mimiviruses with humans, especially regarding pneumonia cases [50-57].

## Viral particle structure, genome and gene expression

**Figure 1:**
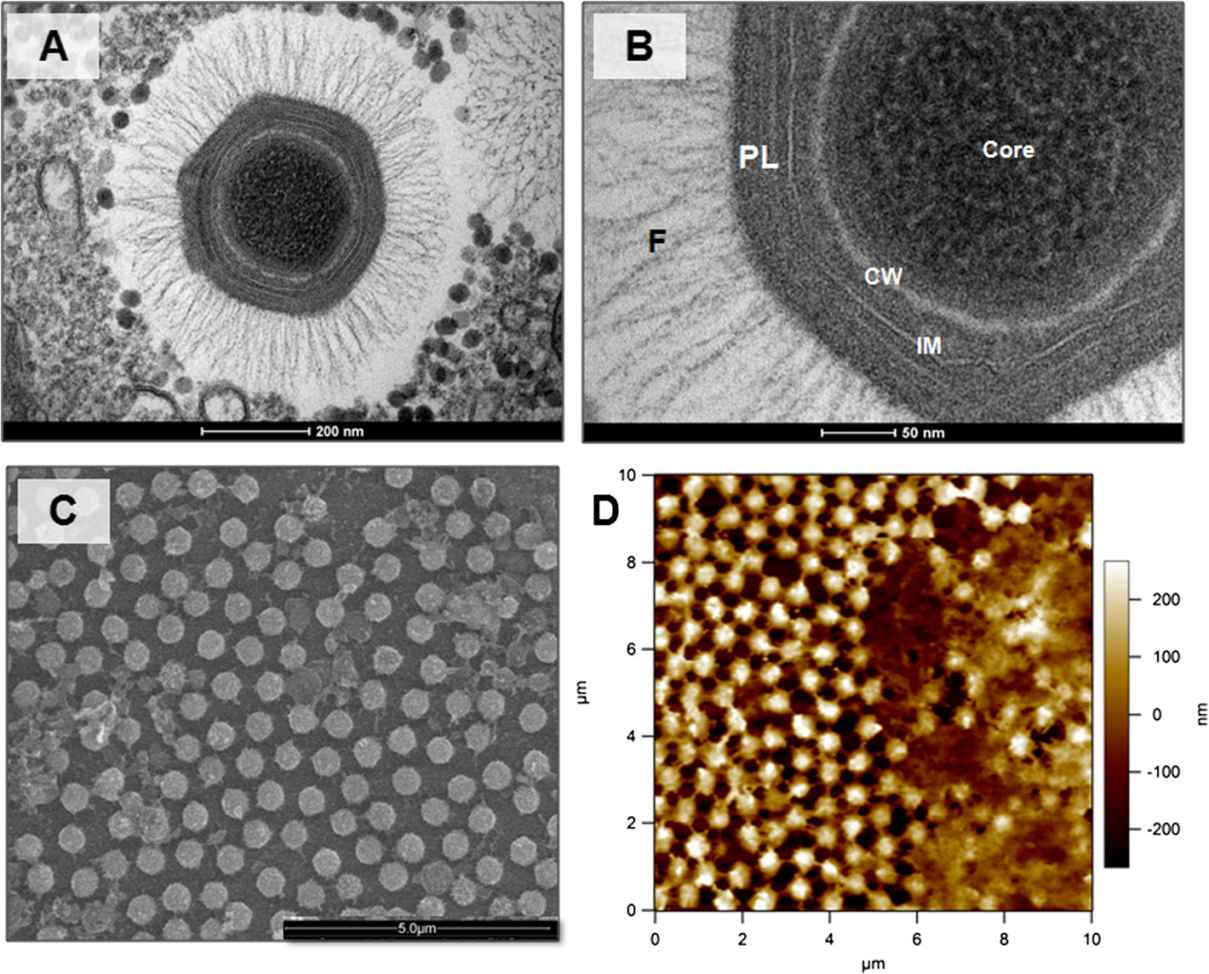
**Mimivirus particle visualized by different microscopy methods.** Transmission electron microscopy of APMV showing the complete particle **(A)** and a zoom **(B)**, highlighting the fibrils **(F)**, the capsid protein layers (PL), the internal membrane (IM), and the core wall (CW) that protects the viral genome and early factors. **(C)** and **(D)** show mimivirus isolates under scanning and atomic force microscopy, respectively. Scale in **(D)** represents the sample depth and size.

**Figure 2:**
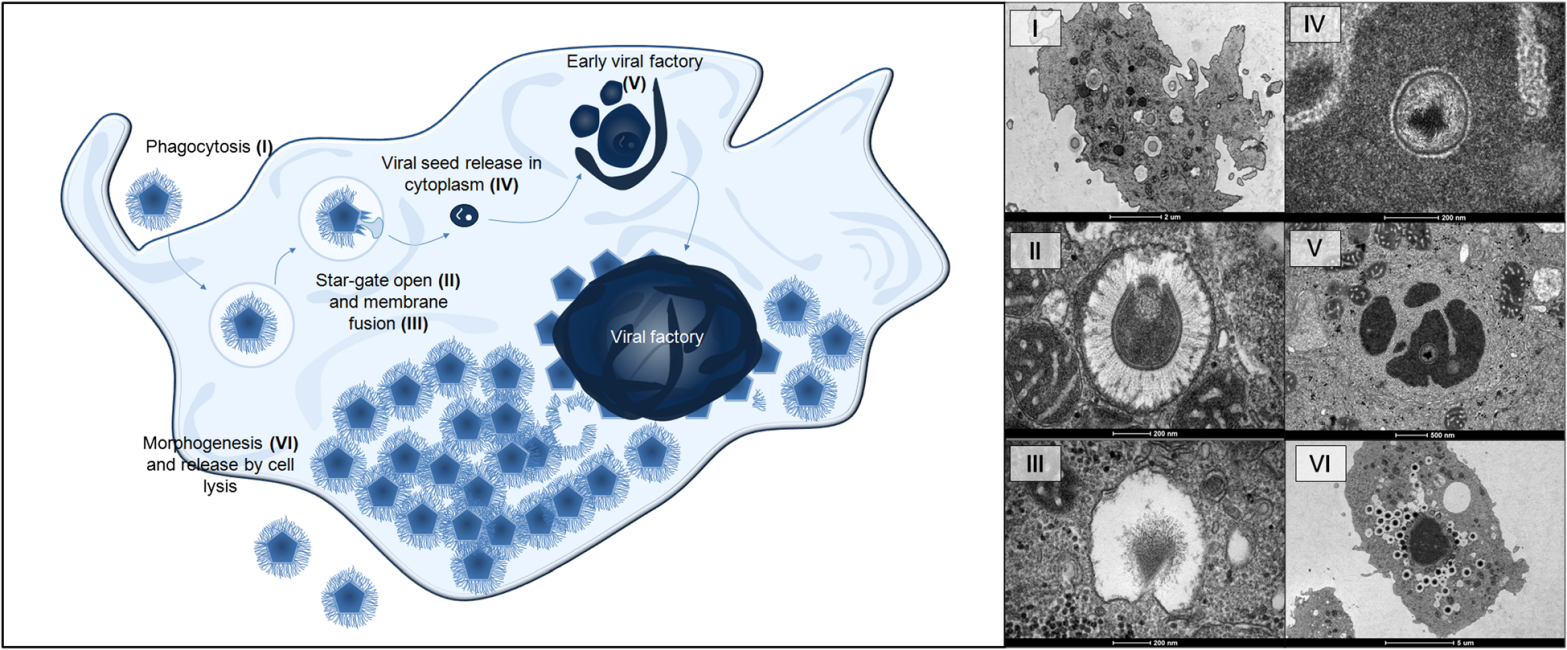
**Mimivirus replication cycle in amoebas. (I)** Phagocytosis. **(II)** Virus entry into a phagosome, followed by star-gate opening and viral membrane fusion **(III)**. **(VI)** Viral seed is released in the amoeba cytoplasm and gives start to an early viral factory **(V)**. After few hours, the viral factory grows and orchestrates the morphogenesis **(VI)** of the viral progeny, which are released by cell lysis. At the right, transmission electron microscopy of APMV at its different steps of the replication cycle.

The remarkable size of mimiviruses and their peculiar features make them unique among viruses. First of all, their enormous size of approximately 700 nm lets them be retained on 0,2 μm filters [5] (Figure 1-A to D). APMV particles do not have an outer envelope, but fibers of approximately 120 nm can be associated with the capsid [58,59] (Figure 1-A and B). These fibers, still under investigation, may be involved in viral adsorption to substrates. APMV encodes enzymes capable of synthesizing polysaccharide complexes found in bacterial lipopolysaccharide (LPS) and/or peptidoglycan [60-63]. These polysaccharides form the viral particle’s outer layer, in which the fibers are embedded, and they may also serve as a phagocytosis stimulus [58-63]. APMV has a pseudo-icosahedral symmetry, with a complex pentagonal face region named stargate [60,63]. This stargate form is a star-shaped projection from which the viral genome is released, and it can be important during early stages of the viral replication cycle [60,61,63,64] (Figure 2).

The particle also contains internal membranes surrounding the genome, likely acquired from endoplasmic reticulum [62] (Figure 1-A and B). The number of lipid membranes inside mimivirus particles is still under debate and investigation. At least three protein layers surround the internal membrane, and the fibrils are projected from the external layer in a tussock-like organization [62] (Figure 1-B). Inside the APMV membrane, a core wall envelops the viral DNA [62,63]. Some authors conjecture that APMV’s unique particle structure may reflect horizontal gene transfer (HGT), especially amongst organisms that share the intracellular environment of amoeba, though adaptative convergence should also be considered. The APMV genome is found in association with a fibrous matrix, resembling the structure of most eukaryotic genomes; the genome release by the stargate mechanism is similar to genome releases in certain bacteriophages, and the peptidoglycan matrix surrounding the external fibers is similar to that found in certain bacteria [63].

This large and complex particle structure seems to be vital for viral stability and genome integrity under adverse environmental conditions [65-67]. The APMV genome consists of a double-stranded DNA molecule of approximately 1,2 Mb that encodes approximately 1000 proteins, many of them still uncharacterized or having functions never observed before in other viruses [5,68]. Four main groups of ORFs can be delineated in the APMV genome: (i) Megavirales core genes; (ii) genes involved in lateral gene transfer; (iii) duplicated genes; and (iv) ORFans [69]. Some APMV encoded proteins are involved in protein translation, DNA repair, cell motility and membrane biogenesis [68-72]. Many of these proteins are likely non-essential for viral replication, but may possibly increase viral fitness. Some APMV genomic sequences exhibit little or no homology with any other known nucleotide sequences in the current databases, and thus are called ORFans [69-72]. The presence of genes encoding proteins involved in protein translation, such as the amino-acyl tRNA synthetase (aaRS) and translation factors, confers to APMV a degree of independence from their host cell machinery for genome replication [73,74].

No previously described viral genomes have shown a genetic arsenal that is able to encode elongation factors, such as tRNA and aaRS; those viruses instead must directly rely on host elongation factors. A substantial proportion of the Mimivirus, and Marseillevirus, ORFs have homologs in bacteria, archaea, eukaryotes or viruses. The large amount of chimeric genes in these viral genomes may have resulted from acquisitions by lateral gene transfer, implying sympatric bacteria and viruses with an intra-amoebal lifestyle [69].

The genomes of APMV and other mimiviruses have a high adenine-thymine (AT) content, with their most frequent codons being AAA (lysine) and AAT (asparagine). Both the codon and amino acid usages of mimiviruses are highly dissimilar to those of their amoebal host, *Acanthamoeba castellanii,* and instead are correlated with the high adenine and thymine (AT) content of the mimivirus genomes [73,75]. Additionally, it has been demonstrated that the Leu(TAA)tRNA present in several mimivirus genomes, and in multiple copies in some viral genomes, may complement the amoebal tRNA pool and may help accommodate the AT-rich viral codons [75]; remarkably, the genes most highly expressed at the beginning of the mimivirus replicative cycle have a nucleotide content more adapted to the codon usage in *A. castellanii*. Recently, an interesting study evaluated genomic alterations in APMV maintained for 150 successive passages in an axenic amoebal culture in an allopatric system. It was shown that the genome was reduced in size and morphological changes occurred in the viral particle [76]. In the allopatric system, there was no competition with other intra-amoebal micro-organisms, including bacteria and other viruses; nor were there major sources for gene gain and replacement from those micro-organisms. The passaged APMV had large deletions towards the APMV genome extremities and gene losses that were associated with: loss of fibers and their glycosylation; decreased viral ability to associate with virophages; and decreased particle antigenicity, likely due to fiber loss [76]. Excluding large deletions, 77% of the APMV genes remained intact after 150 passages in amoeba, 23% had variability and 10% were predicted to have inactivated. A majority of these inactivated genes had been previously described as weakly transcribed in APMV, prior to the laboratory culture under allopatric conditions [68,77]. In contrast, most of the genes highly transcribed before this laboratory culture were not inactivated. The major loss of weakly transcribed and weakly expressed genes in allopatric conditions suggests that the virus tends to lose or degrade its useless genes in a Lamarckian evolutionary process [77]. The loss can also be explained by the fact that DNA repair is most common in actively transcribed regions, so it is expected that genes exhibiting lower transcriptional levels undergo more changes [76,77].

## Viral replication cycle

Although APMV is also unique in its replication cycle, there is a resemblance to the poxvirus replication cycle [78] (Figure 2). It has been demonstrated that APMV enters amoebas of the *Acanthamoeba* genus through phagocytosis (Figure 2-I), and the initiation of the replication cycle is marked by a typical eclipse phase, in which viral particles are not viewed in the cell [5,39]. In the early stages of replication, phagocytosed viral particles can be detected within phagosomes inside the host cell until the star-gate channels in the viral capsids (Figure 2-II) open, which is followed by membrane fusion (Figure 2-III) and release of viral seeds containing the genomes into the cytoplasm of the host cell (Figure 2-IV) [78]. DNA replication occurs exclusively in the cytoplasm, although it cannot be considered totally independent of the host nucleus because nuclear factors required for replication might participate in the process. It has been demonstrated that multiple vesicles start to appear in the cytoplasm approximately 2 hours after infection [5,78]. Their origins are unknown, but it is suspected that they are derived from the nuclear membrane or endoplasmic reticulum, and these vesicles seem responsible for nuclear factor transportation to the viral factories [78,79]. Atomic microscopy of infected amoebas revealed that the nuclear morphology does not change during the APMV infection cycle [80]. Following uncoating, the genome stabilized in viral seeds initiates the viral factories (Figure 2-V factory in formation; 2-VI mature factory). In these factories, viral DNA undergoes replication and transcription, and the DNA is prepared to be packaged in procapsids through a non-vertex portal, a transient aperture centered at an icosahedral face distal to the DNA delivery site, suggesting a pathway reminiscent of DNA segregation in bacteria [63,64,78,79].

The transcription occurs in a temporal manner: early, intermediate and late stages [80]. Interestingly, it was demonstrated that mimivirus gene promoters exhibit an unprecedented conservation among all eukaryotes [80]. After the expression of late genes, there is an increase in viral factories and structural proteins are synthesized, initiating the process of viral morphogenesis, followed by packaging of capsids with DNA (Figure 2-VI). During morphogenesis, membrane generation is accompanied by the assembly of icosahedral viral capsids, a process involving the hypothetical major capsid protein L425 that acts as a scaffolding protein [33]. An assembly model was proposed explaining how multiple mimivirus progeny can be continuously and efficiently generated. There is a high accumulation of virions in final stages of morphogenesis [78] (Figure 2-VI). It was also demonstrated in an interesting study, that professional phagocytes such as vertebrate monocytes and macrophages are permissive for APMV replication, becoming infected via phagocytosis that leads to productive infections [39]. Ultrastructural analysis showed that protrusions were formed around the entering virus, suggesting that macropinocytosis or phagocytosis was involved in APMV entry. Reorganization of the actin cytoskeleton and activation of phosphatidylinositol 3-kinases were required for APMV entry [39]. However, although it has been shown that APMV is able to interfere with the IFN system in vertebrate cells, further studies are necessary to understand the mechanisms involved in viral replication in vertebrate phagocytes [49].

## The role of giant viruses in aquatic ecosystems

The isolation of giant viruses from many different specimens, ranging from environmental samples to unicellular eukaryotic green algae and even vertebrates, reveals their ubiquitous presence on this planet. To date, we have isolated many giant viruses from aquatic environments in Brazil, especially from urban lagoons and acidic rivers, suggesting an association between a high degree of organic matter and giant virus detection [81] (unpublished data). However, discovery of mimiviruses raises questions about their ecological and evolutionary roles, especially in oceans. Carbon transfer and nutrient recycling are important biogeochemical processes that deeply involve marine zooplankton and phytoplankton [82,83]. Viruses are key regulators of these processes, due to their ability to infect and kill these populations, but the full extent of their role is still unknown [82,83]. The discovery of giant viruses in fresh and salt water provoked intense debate about the ecology of these viruses in aquatic systems, as well as their roles in structuring protist populations and even in gene exchange. In recent studies, it became evident that giant viruses can infect host protists, as in the case of the *Cafeteria roenbergensis virus* infecting marine unicellular chlorophyll flagellates [84]. It was suggested that the APMV group also contains closely related viruses capable of infecting phytoplankton, and these viruses groups phylogenetically with certain viruses of unicellular and multicellular algae [85]. Metagenomic studies, conducted in 2005 during the Global Ocean Sampling expedition in the Sargasso Sea, demonstrated through phylogenetic analysis that DNA viruses that are evolutionarily close to mimivirus exist in nature [10,86,87].

Several mimivirus-like sequences were identified, suggesting that these viruses are abundant in the marine environment. This key finding suggests that further studies of these genomic sequences can reveal the diversity of these DNA viruses and their possible roles in the evolution of eukaryotes [86]. To a lesser extent but similar to the interaction of phages and bacteria, prokaryotes that are lysed by the multiplication of marine mimiviruses could result in increased dissolved carbon and nutrients in surface waters, which might reduce sedimentation, promote microbial growth and impact local communities.

## Giant viruses and the contribution to evolution knowledge

Canonically, it is considered that the genomes of most viruses do not contain sufficient information to support their classification into a domain of life. However, with the discovery of APMV and other giant viruses, this topic is being debated [24,26,88]. Giant viruses have genes that are common to the three classical Domains of life: *Archaea*, *Bacteria* and *Eukarya*, including genes involved in information storage and processing. For some researchers, this phenomenon puts them in the same definition of life that is assigned to those Domains [26]. Some APMV genes are notable due their hypothetical evolutionary importance. Recent studies have indicated the existence of a host-independent glycosylation system in APMV, likely acquired very early during evolution [89]. Collagen, one of the most abundant proteins in living cells, is found in APMV and undergoes a new type of glycosylation, showing for the first time that post-translational collagen modifications are not restricted to the canonical domains of life [90]. These results indicate that mimiviruses may have contributed to the evolution of collagen biology [90]. Genes coding for proteins involved in the replication and repair of DNA, such as DNA polymerase B and topoisomerase II, and genes for the thymidine synthetase enzymes involved in the biosynthesis of DNA oligonucleotides, are typical of eukaryotic cells but are also found in giant viruses [73]. A phylogeny based on these proteins results in an exclusive clade for the giant viruses, distinct from *Eukarya*, *Bacteria* and *Archaea*[26].

Analysis of the transcription factor II B, absent in *Bacteria*, suggests that it is highly conserved and forms a clade as old as *Eukarya* and A*r*chaea itself [88]. Analysis of the aaRS genes reveals similarity between *Mimiviridae*, *Amoebazoa* and *Eukarya*. It supports the possibility that these genes may have been transferred from a viral ancestor to amoebae, suggesting that these exchanges are common [88]. However, this specific gene has been described as involved in a lateral gene transfer. These intriguing results led some authors to question the comprehensiveness of phylogenetic trees based on analysis of ribosomal RNA because they exclude viruses and do not seem sufficient to represent all forms of life and to propose a fourth domain of life based on phylogenetic and phyletic analyses of informational genes [24,26,88]. The current classification, based on patterns of similarity of ribosomal RNA, is a prejudiced approach because it excludes viruses from the living organisms, as viruses do not harbor ribosomal genes. However, the proposition of a fourth Domain of life to accommodate giant viruses is still much in debate. Some authors believe that the fourth domain may be artifactual, due to compositional heterogeneity and homoplasy, and the use of genes possibly acquired by the viruses from their eukaryotic hosts by horizontal gene transfer (HGT) [88].

## Virophages

The isolation of *Acanthamoeba castellanii mamavirus* (ACMV) led to the discovery of one of the most intriguing and differentiating features of the *Mimiviridae* family: its close association with other small viruses called the *Sputnik virus. Sputnik virus* was first identified as a satellite virus [11]. Its replication, associated with the *Mimivirus* factories inside amoebas, resembled satellite viruses that affect animals and plants. However, *Sputnik virus* replication appears to impair the normal morphogenesis and production of *Mimivirus,* a process closer to true parasitism than to the previously known satellite viruses. From a biological view, the infection with *Sputnik virus* results in ~ 70% reduction of the cytopathic effect of the giant viruses in amoeba and leads to formation of some atypical viral forms, in a way never described for traditional satellite viruses [11,12]. This discovery led to the creation of the term “virophage”, which means a virus able to ‘infect’ other viruses [11,12]. *Sputnik virus* is an icosahedral virus 50 nm in diameter and a genome of 18 kb, which contains a mosaic of genes related to bacteriophages, other viruses and amoebae. Its genome is circular double-stranded DNA that is hypothetically able to encode 21 proteins, some of which have no detectable homologues in current databases of nucleotide sequences [11,12].

Recently, we have isolated Rio Negro virophage associated with Samba virus, an APMV strain from the Brazilian Amazon [81]. Other described virophages are parasites of the giant viruses (phycodnaviruses and mimiviruses) [32] and might control the dynamics of Antarctic alga species. Mavirus is a virophage that parasitizes the giant *Cafeteria roenbergensis virus*[91]. On the basis of genetic homology, Mavirus likely represents an evolutionary link between double-stranded DNA viruses and Maverick/Polinton eukaryotic DNA transposons [91]. It has been shown that giant viruses may even have virophages inside their own mobile genetic elements. A 2013 study of the mobilome of *Lentille virus* revealed that a virophage, *Sputnik 2*, is part of this genetic element [14]. It was observed by FISH that the virus and its virophage replicate in the same viral factories and are detected in the cytoplasm of the host cell, suggesting that *Sputnik 2* was integrated into the *Lentille virus* genome [14]. This was confirmed by genome digestion of these viruses, followed by Southern blotting and 2D gel analysis. *Lentille virus* was sequenced on different platforms, and a sequence related to *Sputnik2* was integrated in its genome, thus suggesting that *Sputnik2* is a provirophage. These analyses also revealed that *Lentille virus* (and a small fraction of *Sputnik2*) contained an extrachromosomal DNA rich in CG that was called a transpoviron (an equivalent of transposon in viruses from our point of view), which can undergo recombination with *Sputnik2* and many other organisms [14]. Phylogenetic analysis of the virophages and related genetic elements is compatible with the concept of a network-like evolution in the virus world and emphasizes multiple evolutionary connections between bona fide viruses and other classes of capsid-less mobile elements. Thus, giant viruses appear to present a complex mobilome, which could contribute to gene exchanges that are common in these viruses. Further investigation of these elements will possibly lead to new discoveries, including novel classes of mobile elements, thanks to the diversity and complexity of giant viruses and their virophages [92].

## Clinical significance

Hospitalized patients are a risk group for nosocomial infections, including those caused by amoeba-associated pneumonia agents [54,93]. APMV is a putative pneumonia agent and studies associating this virus with human pneumonia cases are still under investigation [51,54,55,94-97]. APMV genetic material (once) and antibodies against APMV have been detected in samples from patients who had pneumonia without any known cause (bacterial, viral or fungal); these patients came from different locations and were studied by different research groups, lending strength to the possible role of APMV as a pneumonia agent [54-57,98]. The genetic diversity among the *Mimiviridae* members needs to be considered when interpreting negative PCR tests in several studies because the diversity may have impaired detection of APMV-related DNA [29]. An animal model for mimivirus pneumonia studies has been proposed. When various routes of infection were tested, the intracardiac infection route induced pneumonia in C57/B6 mice [45]. Although this model does not exactly simulate the hypothetical natural route of mimivirus infection, it was possible to observe histopathological evidence of acute pneumonia, isolate the virus and detect antigens by indirect immunofluorescence assay [45].

The first studies in which a giant virus was successfully isolated from a human specimen were published in 2013 [50,52]. In a first study, a total of 196 samples from patients were collected in Tunisia between 2009 and 2010; virus was detected by the formation of plaques in monolayers of amoeba grown on agar plates [52]. The isolated virus (LBA111) was found in a sample obtained from a 72-year-old patient with pneumonia. Serology and real time PCR confirmed the presence of a giant virus in this sample. LBA111 has a similar morphology to other mimiviruses, with a size of approximately 560 nm and a genome of 1,23 Mb. Western blot analysis showed positive immunoreactivity of patient sera against specific proteins of both APMV and LBA111 [52]. In another study, Shan virus was isolated from a stool sample collected from a Tunisian patient with pneumonia [50]. Metagenomic analysis of a human stool sample revealed the presence of sequences similar to those of giant viruses. From these samples, a virus was isolated and named Senegalvirus [53]*.* Its characteristics linked it to the *Marseilleviridae* family, representing the first detection of a marseillevirus from a human sample [53]. Additionally, a blood sample from an apparently healthy donor revealed the presence of Marseillevirus-like DNA; antigens from this virus were detected, it was visualized by microscopy and it grew in human T cell lymphocytes [46].

The potential clinical relevance of mimivirus can also be analyzed by studies involving the host-virus relationship. In 2008, it was observed that APMV is internalized by professional phagocytic cells such as macrophages, but not by non-phagocytic cells like fibroblasts, epithelial or neuronal cells [39]. This suggests that these professional phagocytic cells can be targets for APMV replication in humans. Analysis of ‘infected’ macrophages revealed that viral DNA increased following infection, and APMV was seem as cytopathogenic for these cells [39]. Recently, a study investigated whether human peripheral blood mononuclear cells (PBMC) can recognize APMV presence by measuring IFN induction; whether APMV can replicate in these cells; and whether replication of the virus is affected by treatment of cells with IFN type I [49]. The results showed that APMV is able to replicate in human PBMC and induce type I IFN in these cells and that it inhibits some IFN-stimulated genes (ISG) by a mechanism that is independent of viroceptors and STAT dephosphorylation [49]. It was also seen that APMV is resistant to the antiviral action of IFN alpha2 (IFNA2), but is sensitive to the antiviral action of IFN beta (IFNB1). These results not only confirm that APMV can indeed replicate in vertebrate phagocytes but also show that it is recognized by the innate immune system and that it is likely able to at least partially evade that system [49]. This interaction is most likely the result of co-evolution between APMV and vertebrate hosts, and it is strong evidence of an ancestral relationship between these organisms [49]. Recently, mouse exposure to mimivirus collagens was shown to induce anti-collagen antibodies that also targeted mouse collagen type II, and the exposure was associated with T-cell reactivity to collagen and joint inflammation [99]. Furthermore, a serologic study found reactivities to the mimivirus collagen protein L71 in 22% of rheumatoid arthritis patients, compared to 6% of healthy-subjects. Thus, while clinical studies involving mimivirus are still a growing field, research over the past few years is strengthening the idea that mimiviruses, which are broadly distribution in our biosphere, not only have an environmental impact but are also involved in human health.

## Viral resistance to chemical and physical biocides

The probable importance of APMV as a human pathogen in hospital environments has led to the need for investigating the virucidal activity of chemical biocides used for disinfection in hospitals. In a study performed in 2012, it was shown that APMV is especially resistant to alcohols but is sensitive to the action of active chlorine and glutaraldehyde, and it is able to remain stable on inanimate surfaces for 30 days, even in the absence of organic matter; this highlights the need for best strategies to control this putative pneumonia agent in hospital environments [69]. Amoebae, the hosts of several giant viruses, may act as biological platforms in the spread of pathogens such as *Legionella*, *Parachlamydia*, *Mycobacterium* and also APMV, representing a public health concern. In 2013 we investigated the APMV survival in certain adverse conditions when present in the intracellular environment of *A. castellanii*[100]. It was found that when APMV is inside an amoebal cell subjected to UV irradiation, heat or exposure to different chemical biocides, it remains more stable, showing that these hosts can act like natural bunkers for APMV, increasing its resistance to the viral agents used to disinfect hospital environments [100]. In addition, *Acanthamoeba* spp. may represent a training field for human pathogens, as several micro-organisms resisting these amoebae were concurrently found to resist human macrophages [40].

## Giant viruses publication indicators

**Figure 3:**
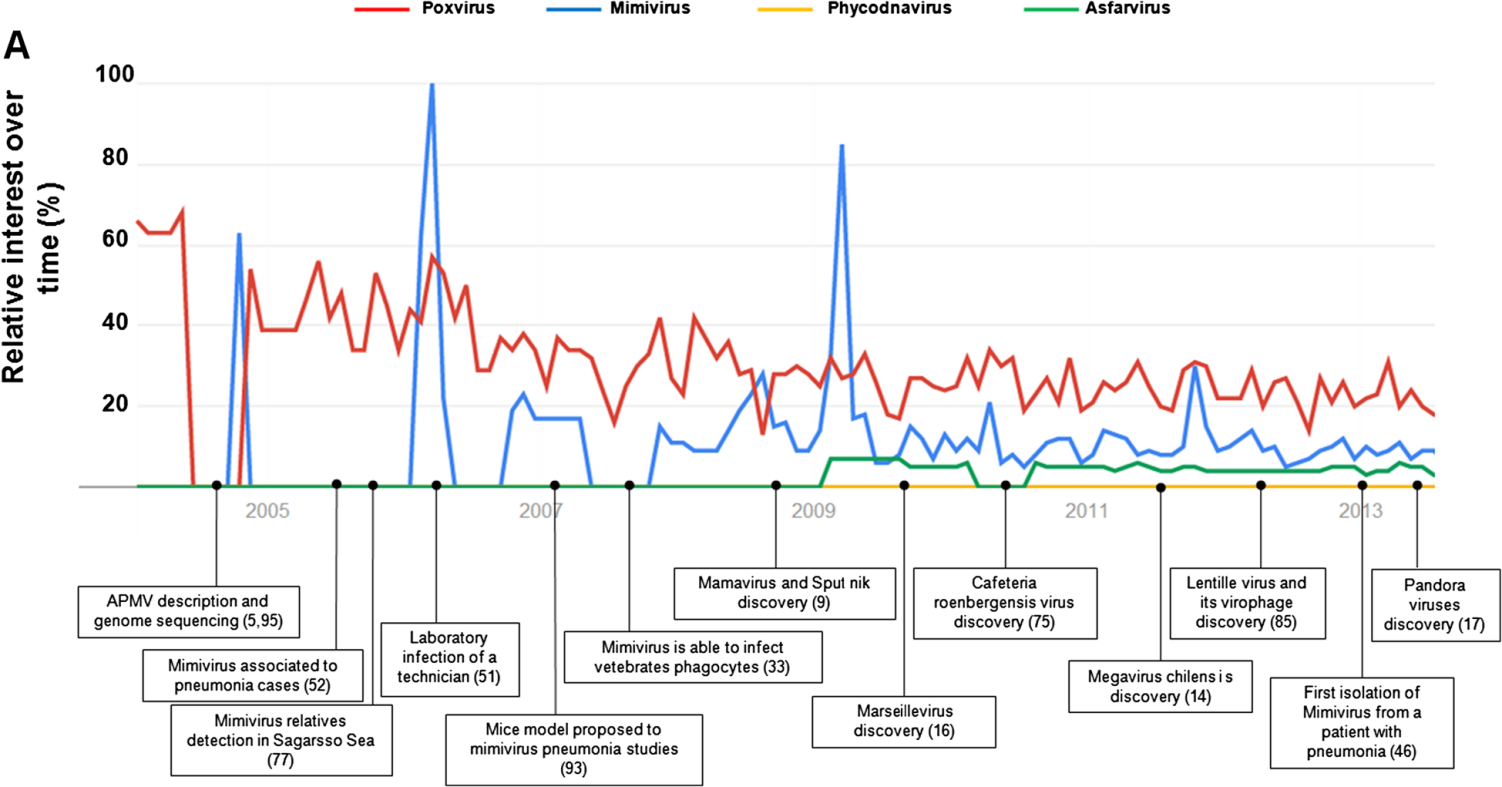
**Timeline highlighting some hallmarks of mimivirus and related topics.** Interest on the subject “Mimivirus”, “Poxvirus”, “Phycodnavirus” and “Asfarvirus” as a research terms in Google Search, over time. The incidence of the research for these subjects in Google Search was obtained by the use of Google Trends tool (http://www.google.com/trends/). The graphics depict relative interest over time (%). The values are in comparison to the highest number of searches at a given occasion (see “help” in Google Trends website). Google trends show available data since 2004. A timeline was set in the bottom of the figure, highlighting some hallmarks of mimivirus and related topics.

**Figure 4:**
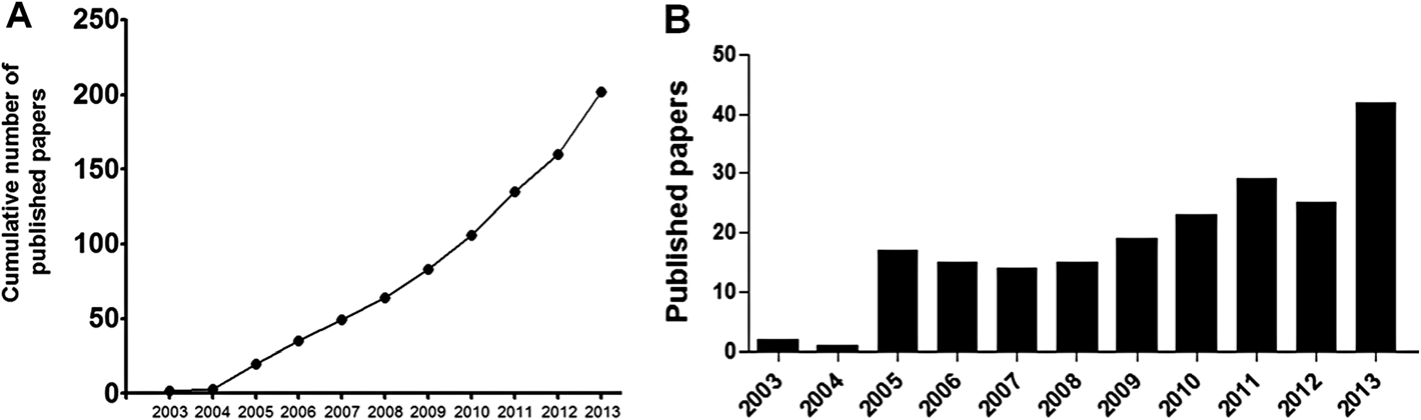
**Cumulative number of publications relating to mimivirus and “mimivirus”/”giant viruses” over time since its discovery in 2003 (http://www.ncbi.nlm.nih.gov/pubmed)”.(A)** Cumulative number of publications relating to mimivirus over time. The subject was researched on Pubmed page (http://www.ncbi.nlm.nih.gov/pubmed), demonstrating the growing number of papers related to the virus since its discovery in 2003. **(B)** Published “mimivirus”/”giant viruses” papers per year since 2003, also according to the Pubmed website.

An indicator of the growth of related research fields concerning giant viruses is the recent increase in the number of publications about APMV and other giant viruses. In 2013, the number of papers related to these fields was 21-fold greater than to the number of papers published in 2003, the year of APMV discovery (Figures 3 and 4). Interest in APMV also increased in internet search tools such as Google Search (Google Trends) over the years, with peaks in 2004, 2006 and 2009 (Figure 3) that most likely are related to mimivirus research hallmarks, such as its discovery, its association to virophages and studies describing APMV as a putative pneumonia agent [5,11,57,73] (Figure 3). Currently, mimiviruses are the second group amongst NCLDVs most searched in the Google platform, and, on some occasions, even overcomes the well-known poxviruses (Figure 3).

## Conclusions

As described, APMV and other giant viruses have emerged as a fascinating line of research. Each discovery regarding mimiviruses has overwhelmed scientists from different areas of expertise, which may explain why so many outstanding publications are multidisciplinary. The future of mimivirus studies might go beyond the description of bigger and more complex viruses but also may contemplate deep structural, genetic and evolutionary studies. With all this knowledge, we expect it will be possible to understand the exact role of mimiviruses in environmental dynamics and their importance as etiological agents of pneumonia in humans and other animals.

## Competing interests

The authors declare that they have no competing interests.

## Authors’ contribution

JSA, FPD, LCS, GMA, PVB, PC, BS and EGK wrote the manuscript. PVB performed analysis in Google Trends and Pubmed. FPD performed electron microscopy analysis. JSA designed the figures. All authors read and approved the final manuscript.
